# Light Spectral Composition Influences Structural and Eco-Physiological Traits of *Solanum lycopersicum* L. cv. ‘Microtom’ in Response to High-LET Ionizing Radiation

**DOI:** 10.3390/plants10081752

**Published:** 2021-08-23

**Authors:** Ermenegilda Vitale, Luca Vitale, Giulia Costanzo, Violeta Velikova, Tsonko Tsonev, Palma Simoniello, Veronica De Micco, Carmen Arena

**Affiliations:** 1 Department of Biology, University of Naples Federico II, Via Cinthia, 80126 Napoli, Italy; ermenegilda.vitale@unina.it; 2 National Research Council, Department of Biology, Agriculture and Food Sciences, Institute for Agricultural and Forestry Systems in the Mediterranean, P. le E. Fermi 1, 80055 Portici, Italy; 3 Department of Agricultural Sciences, University of Naples Federico II, Via Università 100, 80055 Portici, Italy; giulia.costanzo2@unina.it (G.C.); demicco@unina.it (V.D.M.); 4 Institute of Plant Physiology and Genetics, Bulgarian Academy of Sciences, Acad. G. Bonchev Str., bl. 21, 1113 Sofia, Bulgaria; violet@bio21.bas.bg; 5 Institute of Biophysics and Biomedical Engineering, Bulgarian Academy of Sciences, Acad. G. Bonchev Str., bl. 21, 1113 Sofia, Bulgaria; ttsonev@bio21.bas.bg; 6 Department of Science and Technology, University of Naples Parthenope, Via Acton n. 38-I, 80133 Napoli, Italy; palma.simoniello@uniparthenope.it; 7 BAT, Interuniversity Center for Studies on Bioinspired Agro-Environmental Technology, 80055 Portici, Italy

**Keywords:** indoor cultivation, heavy ions, light quality, photosynthesis, Rubisco expression

## Abstract

This study evaluated if specific light quality (LQ) regimes (white fluorescent, FL; full-spectrum, FS; red-blue, RB) during plant growth modified morphological and photosynthetic traits of *Solanum lycopersicum* L. ‘Microtom’ plants irradiated at the dry seed stage with 25 Gy ^48^Ca ions (IR). The irradiation reduced plant size while it increased leaf dry matter content (LDMC) and relative water content (RWC) compared to the control. FS and RB light regimes determined a decrease of plant height and a rise of RWC compared to FL plants. The irradiation under FS and RB regimes favoured the development of dwarf plants and improved the leaf water status. Under the FL regime, irradiated plants showed reduced photosynthesis and stomatal conductance. The opposite behavior was observed in RB irradiated plants in which gas exchanges were significantly stimulated. RB regime enhanced Rubisco expression in irradiated plants also inducing anatomical and functional adjustments (i.e., increase of leaf thickness and incidence of intercellular spaces). Finally, ^48^Ca ions did not prevent fruit ripening and the achievement of the ‘seed-to seed’ cycle, irrespective of the LQ regime. Overall, the present study evidenced that RB light regime was the most effective in optimising growth and photosynthetic efficiency of ‘Microtom’ irradiated plants. These outcomes may help to develop proper cultivation protocols for the growth of dwarf tomato in Controlled Ecological Life Support Systems (CELSS).

## 1. Introduction

The cultivation of crops in controlled conditions guarantees food production throughout the year, overcoming the limitations of environmental constraints. In the last years, the utilization of innovative lighting sources for plant growth has increased exponentially. In particular, the use of light-emitting diodes (LEDs) technology allowed us to create suitable light environments for indoor cultivation by selecting specific wavelengths able to elicit plant-exclusive photomorphogenic, biochemical or physiological responses [1,2,3]. The manipulation of the light spectrum is a widely applied methodology to improve plant photosynthesis and secondary metabolite production, including bioactive compounds beneficial for human health.

Even if the plant response to different light quality treatments is species-specific, some outcomes may be comprehensive. Generally, the red (R) and blue (B) wavelengths are the most effective for photosynthesis and drive morphogenesis since the early developmental stages [4]. The B wavelength perceived through cryptochromes controls many processes such as stem elongation, phototropism, chloroplast movement within cells, stomatal opening, and elicits the biosynthesis of secondary metabolites such as flavonoids [5,6]. The R light contributes to photosynthetic apparatus development and influences morphogenesis.

Nevertheless, even if monochromatic R or B lights do not fully satisfy the normal plant growth requirements, the lack of one or an unbalanced proportion between R and B produces several photosynthetic inefficiencies. A higher percentage of B compared to R often results, in lower photosynthetic rates and biomass production, while the absence of B induces anomalies in growth, such as a stretched, elongated appearance and dysfunction of the photosynthetic process [4].

The use of LED technology to modulate light quality is becoming a promising tool in the vertical farming industry and in supporting off-grid agriculture. In a few decades, indoor city farms or vertical farms have become popular for producing healthy food year-round in urban agriculture and harsh climates.

Nowadays, a new challenge is crop production in extreme environments including Space. In this vision, the light quality manipulation would favor an efficient plant growth in Controlled Ecological Life Support Systems (CELSS) developed to support human life in Space. Moreover, in the sight of long-duration Space manned missions, the role of plants in CELSS would be crucial for food production and resource regeneration (i.e., carbon dioxide removal, oxygen production, water purification) on board.

Among the environmental factors in Space, ionizing radiation (IR) is recognized to deeply influence plant growth at molecular, morphostructural and physiological levels, ranging from stimulatory effects at very low doses up to harmful outcomes at higher doses [7].

Generally, the manned Space outposts are shielded against IR and only low, not lethal, doses reach the indoor environment resulting in not lethal effects. Previous experiments have demonstrated that plant irradiation with low doses of carbon, oxygen, argon, or neon ions induced early maturity, high yield and better fruit quality [8,9,10,11,12,13,14].

Recently, Arena and colleagues [15] have demonstrated, for the first time, the effectiveness of ^48^Ca ion in improving eco-physiological, morphological and functional traits of leaves and fruits of tomato plants. In particular, plants sprouting from irradiated seeds at 25 Gy were photochemically more performant and produced fruits richer in antioxidants.

In this paper, we assessed through a multidisciplinary approach if the plant development under specific light quality regimes may enhance in *Solanum lycopersicum* L. ‘Microtom’ plants the positive outcomes of seed irradiation with low doses of Ca heavy ions, not only in terms of morpho-anatomical and biochemical traits but also in optimizing the photosynthetic gas exchanges.

## 2. Results

### 2.1. Effect of Ionizing Radiation and Light Quality on Plant Growth and Leaf Functional Traits

Seed irradiation with Ca ions at a dose of 25 Gy did not affect the germination percentage that reached 100% in both control and irradiated plants. All plants completed the growth–life cycle producing ripe fruits at 120 DAS.

**Figure 1:**
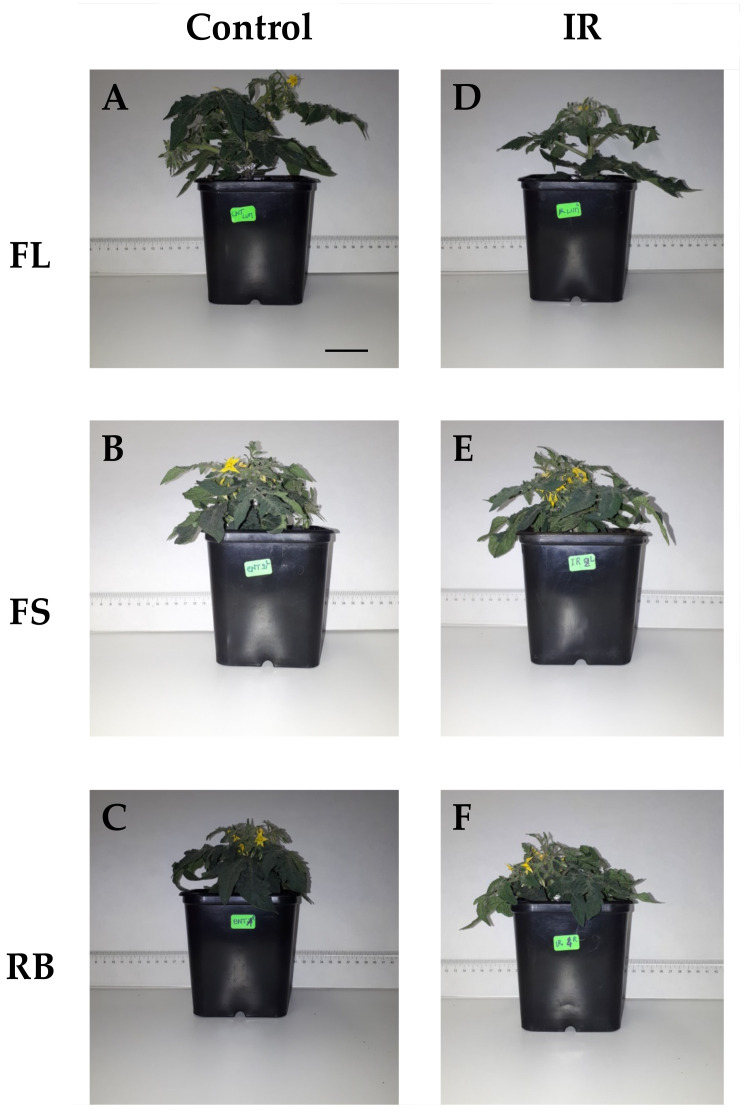
Plants of *S. lycopersicum* L. ‘Microtom’ from non-irradiated control (**A**–**C**) and irradiated (IR) seeds (**D**–**F**) and grown under FL, FS, and RB light quality regimes. Scale bar = 5 cm.

**Figure 2:**
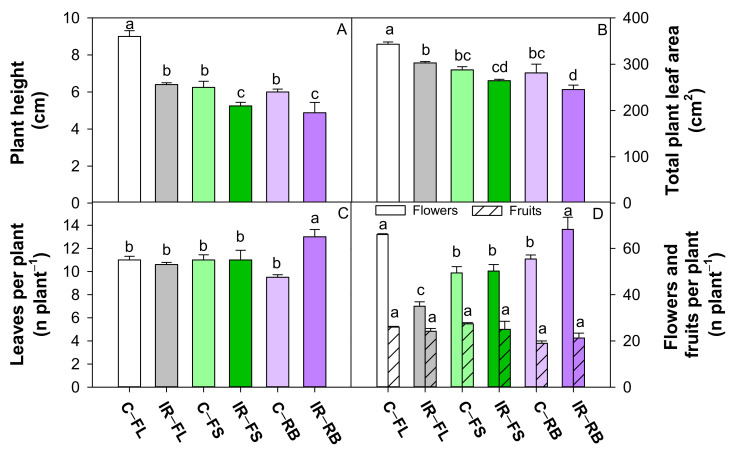
Plant height (**A**), total plant leaf area (**B**), number of leaves per plant (**C**), number of flowers and fruits per plant (**D**) of *S. lycopersicum* L. ‘Microtom’ in non-irradiated control (C) and irradiated (IR) plants grown under FL, FS and RB light regimes. Bars are means ± standard error (*n* = 5). Different letters indicate statistically significant differences according to Student–Newman–Keuls multiple comparison test (*p* < 0.05).

Plant morphological traits were significantly affected by irradiation (IR) and light quality (LQ) as main factors, and by their interaction (IR × LQ) (Table 1, Figure 1 and Figure 2).

Irradiated plants showed a higher (*p* < 0.05) number of leaves but a reduced (*p* < 0.05) number of flowers, total leaf area and plant height compared to control (Table 1). The growth under FS and RB regimes determined a reduction (*p* < 0.001) in plant height and total leaf area compared to FL, irrespective of irradiation. RB plants also showed the highest (*p* < 0.001) flower number and the lowest (*p* < 0.01) fruit number (Table 1) compared to FL an FS light regimes.

The interaction IR × LQ was significant for all growth traits except fruit number (Table 1). As shown in Figure 2, plant height was significantly lower in irradiated plants under FS and RB regimes compared to all the other conditions, with maximum value in C-FL plants. Irradiated plants under FS and RB showed lower TLA than under FL regime, while C-FL plants exhibited the highest TLA among all treatments. In IR-RB plants, the number of leaves was higher than all the other treatments, while the number of flowers was comparable to C-FL plants and significantly higher compared to the other conditions.

IR significantly influenced leaf functional traits with exception of specific leaf area (SLA), while LQ had a significant effect only on leaf area (LA). The interaction IR × LQ was significant for LA and relative water content (RWC) (Table 2).

IR determined the development of leaves characterized by higher (*p* < 0.001) LA, LDMC and RWC (*p* < 0.01) compared to control under all light quality regimes (Table 2). Considering LQ as main factor, FL plants showed higher and lower values (*p* < 0.001) of LA and RWC, respectively, compared to FS and RB. The interactions IR × LQ showed that IR-FL plants were characterized by the highest value of LA, while IR-RB plants exhibited the highest RWC among different IR × LQ combinations.

**Figure 3:**
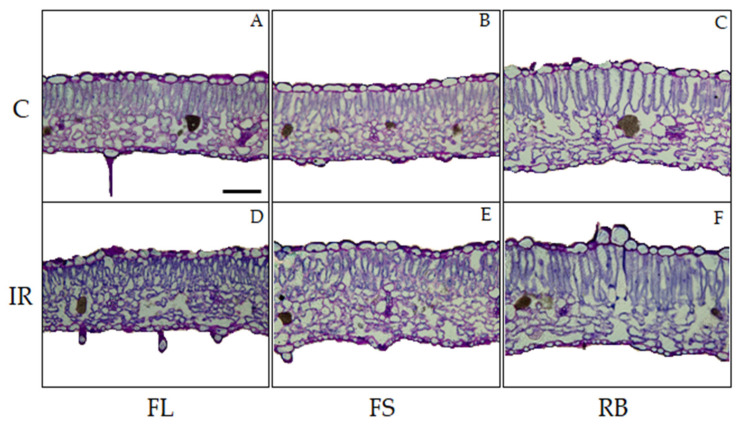
Light microscopy views of cross sections of leaf lamina of *S. lycopersicum* L. ‘Microtom’ in non-irradiated (control) (**A**–**C**) and irradiated (IR) plants (**D**–**F**) grown under FL, FS and RB light regimes. Images are at the same amplification; scale bar = 100 µm.

The light quality regimes and the irradiation treatment did not determine any qualitative change in leaf anatomical features of ‘Microtom’, with leaves showing the distribution of tissues typical of the dorsiventral structure (Figure 3).

IR as main effect did not induce alterations in the analyzed quantitative anatomical traits compared to control (Table 2). Conversely, LQ significantly influenced leaf anatomical traits except for stomata density. In particular, plants grown under RB regime showed leaves with higher incidence of intercellular spaces (IS) (*p* < 0.01) and thicker palisade and spongy tissues compared to FS plants which in turn showed significantly higher values of such parameters than FLs (Table 2, Figure 3).

The interaction IR × LQ was significant only for IS (Table 2). In particular, IR-RB and IR-FL plants showed higher and lower IS values, respectively, compared to plants grown in the other conditions (Table 2).

### 2.2. Effect of Ionizing Radiation and Light Quality on Photosynthetic Gas Exchanges

**Figure 4:**
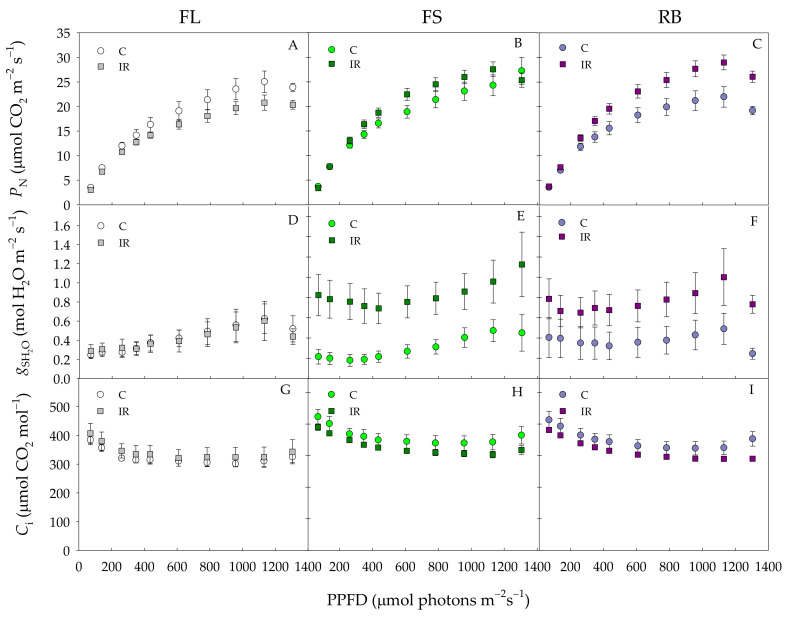
Net photosynthetic rate (P_N_) (**A**–**C**), stomatal conductance (g_s_) (**D**–**F**), intercellular CO_2_ concentration (C_i_) (**G**–**I**) of *S. lycopersicum* L. ‘Microtom’ in non-irradiated control (C) and irradiated (IR) plants grown under FL, FS and RB light regimes. Data are means ± standard error (*n* = 10).

The photosynthetic light response curves of ‘Microtom’ plants are shown in Figure 4.

Photosynthetic rates (P_N_) were similar in control and IR plants up to 400 PPFD under all light growth regimes (Figure 4A–C). Above this value, under FL regime, P_N_ was higher in control than in IR plants, while an opposite behavior was found under FS and RB regimes with P_N_ values higher in IR than control plants.

Stomatal conductance (g_sH2O_) and intercellular CO_2_ concentration (C_i_) did not significantly change between C and IR plants under FL regime (Figure 4D,G). On the contrary, under FS and RB regimes, g_s H2O_ significantly increased IR compared to control plants in the whole PPFD range (Figure 4E,F). C_i_ under both FS and RB regimes declined more in IR than C plants, showing statistically significant differences only for values of PPFD higher than 1200 μmol photons m^−2^s^−1^(Figure 4H,I).

IR did not significantly influence P_Nsat_, I_sat_ or Φ_CO2_ (Table 3). Conversely, LQ significantly influenced both P_Nsat_ and Φ_CO2_ which resulted higher (*p* < 0.01) in FS and RB than FL plants (Table 3). The interaction IR × LQ was significant only for P_Nsat_ and Φ_CO2_. In particular, higher values were reached in IR-FS and IR-RB plants compared to the other treatments (Table 3).

### 2.3. Effect of Ionizing Radiation and Light Quality on Biochemical Compounds, Proteins and Rubisco

The total antioxidant capacity was influenced by LQ as main factor and by the interaction IR × LQ, while polyphenol and flavonoid contents were significantly influenced only by IR and LQ as main factors (Table 4).

**Figure 5:**
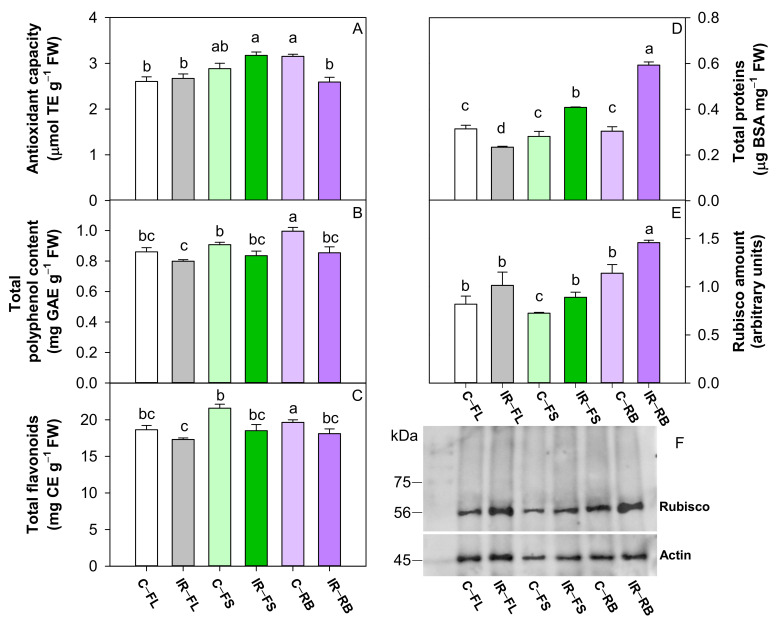
Antioxidant capacity (**A**), total polyphenol content (**B**), total flavonoids (**C**), total proteins (**D**), densitometric analysis and Western blot of Rubisco (**E**,**F**), in leaves of *S. lycopersicum* L. ‘Microtom’ in control (C) and irradiated (IR) plants grown under FL, FS and RB light regimes. Bars are means ± standard error (*n* = 5). Different letters denote statistically significant differences according to Student–Newman–Keuls multiple comparison test (*p* < 0.05).

IR as the main factor reduced (*p* < 0.001) the content of polyphenols and flavonoids compared to the control. Among light regimes, the RB and FS determined a higher (*p* < 0.05) total antioxidant capacity than FL, while RB induced the highest (*p* < 0.01) number of polyphenols and flavonoids among FS and FL (Table 4). C-RB and IR-FS plants showed higher values of antioxidant capacity, while C-RB plants exhibited the highest polyphenol and flavonoid content of all other treatments (Figure 5A–C).

Total proteins and Rubisco amount were influenced by IR and LQ as main factors and by their interaction IR × LQ (Table 4, Figure 5). The irradiation with Ca ions induced a higher (*p* < 0.001, *p* < 0.05) total protein and Rubisco content than controls (Table 4). The growth under RB determined the highest (*p* < 0.001, *p* < 0.01) content of proteins and Rubisco compared to FL and FS regimes (Table 4).

Compared to respective controls, the growth under FL regime reduced the total protein in IR plants and did not change the Rubisco amount; conversely the development under FS and RB regimes increased the total protein and Rubisco in IR plants (Figure 5D,E). In particular, IR-RB plants showed the highest amount of total protein and Rubisco content compared to the other conditions (Figure 5D,E).

### 2.4. Heatmap Analysis

**Figure 6:**
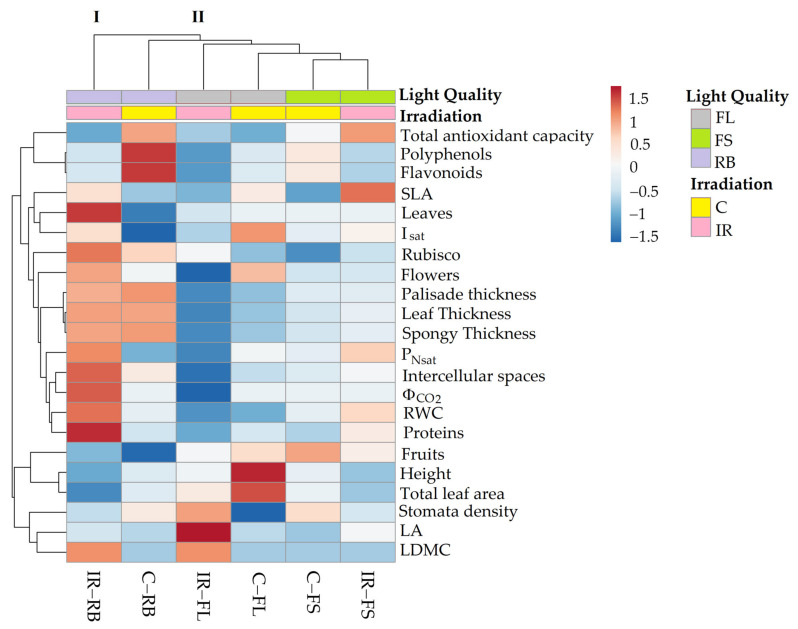
Cluster heatmap analysis of morphological and physiological parameters in non-irradiated control (C) and irradiated (IR) plants of *S. lycopersicum* L. ‘Microtom’ grown under FL, FS and RB light regimes. Numeric differences in the data matrix are displayed as the colour scale with red and blue indicating the increasing and decreasing values, respectively. Parameters are grouped in the rows; sample groups are clustered in the columns by the two independent factors, IR and LQ.

Figure 6 summarizes the morphological and physiological traits of ‘Microtom’ plants in response to ionizing radiation (IR) (Ca 25 Gy) and light quality (LQ) regimes (FL, FS, RB).

The heatmap established two main clusters. The first cluster (I) included IR-RB plants, while the second (II) incorporated the other treatments.

The treatment C-FL induced higher values of plant height, leaf area, flower number and Isat, whereas IR-FL induced greater LA and stomata density.

C-FS plants produced the highest number of fruits, while under IR-FS treatment, higher SLA and antioxidant capacity were observed.

C-RB plants were characterised by a rise in polyphenol and flavonoid content. Finally, the separation of IR-RB plants from the other clusters highlighted that the interplay between Ca-ions 25 Gy and RB light regime significantly affected morpho-anatomical and physiological traits of ‘Microtom’ plants. Indeed, increased RWC, leaf thickness, intercellular spaces, and light-response curve parameters were observed in IR-RB plants.

## 3. Discussion

This study showed that the interplay between ionizing radiation (Ca-heavy ions) and light quality regimes elicits specific structural and ecophysiological responses in ‘Microtom’ plants irradiated at the dry seed target stage, which should be taken into account when designing the cultivation protocols for this species in BLSSs in Space.

The IR represents the principal constraint in the extra-terrestrial environment and may affect plant growth and development at different phenological stages [16]. Exposure to IR generally compromises the germination rate depending on dose, type of radiation, and plant species [15,17,18,19,20]. Our data demonstrate that the irradiation of dry seeds with Ca ions at 25 Gy does not impair the germination process nor prevent the complete life cycle ‘from seed-to-seed’, confirming previous studies which indicate the dry seed as the most radioresistant stage in Microtom plants [15,16,21]. The intrinsic radioresistance of seeds takes into account not only the anatomical structure but also the presence of compounds such as melatonin, and the scarcity of water which may avoid the occurrence of oxidative stress due to radiolysis phenomena, preserving the endosperm from severe damage [15,22,23,24].

Tomato plants sprouted from Ca-ion irradiated seeds showed alterations in some morphological traits such as plant height and total leaf area compared to the control, which determined in irradiated plants a more compact structure and dwarf growth as observed by other authors in several crops [15,25,26,27]. Possible alterations during cell division may induce dwarf growth [18,19]. The “dwarf effect” was emphasized when irradiated plants were grown under specific light quality regimes, and in particular under FS and RB. The high percentage of blue light in FS and RB light regimes, respectively 37% and 40%, might have further influenced the plant size. Indeed, blue wavelengths are recognized to affect cell division and expansion, resulting in reduced stem elongation and leaf area [28,29,30,31,32]. The interplay of heavy ions and light quality regimes significantly affected flower formation. Plants sprouted from irradiated seeds and grown under the FL regime reduced the flower number compared to control according to previous findings on the same tomato cultivar [15]. This negative effect is overturned under FS and RB light regimes, indicating a positive role exerted by red and blue wavelengths on anthesis. It has been previously demonstrated that the B/R ratio <1.0, as in the present study, would promote flowering during tomato seedling growth [29]. The finding that cultivation of ‘Microtom’ at specific wavelengths is capable of counteracting the negative effect of radiation on flowering is promising for Space cultivation insofar as the completion of the seed-to-seed cycle is essential for fruit production onboard and for the production of new viable seeds for the next generations.

Changes observed in leaf functional traits provide further evidence of the high ‘Microtom’ responsivity to both IR and light growth environment. Plants developed from Ca-ion-irradiated seeds showed higher LDMC which positively affected the RWC compared to control. Ca-ions may have induced a more significant investment of photosynthates towards sclerenchyma tissues, increasing the tissue density and facilitating nutrient and water retention [33,34]. At the same time, the higher RWC in plants grown under FS and RB light regimes may depend on the stimulatory effect exerted by the blue light on the root system [35,36]. We hypothesize that a more significant expansion of roots may have positively influenced the water absorption from the soil and, therefore determined the higher RWC in these plants. The combination IR-RB was particularly effective in promoting RWC, suggesting a more performing use of water resource that may result as particularly advantageous, considering that water recycling and saving is essential within the BLSSs.

Qualitative leaf anatomical traits were not altered by irradiation, suggesting that Ca-ions at low doses were insufficient to induce apparent structural modifications [15]. From a quantitative viewpoint, leaf thickness and intercellular spaces were significantly influenced by LQ. More specifically, FS and even more RB light treatments induced the development of thicker leaves characterized by a higher percentage of intercellular spaces. The increase in the incidence of intercellular spaces might be in part responsible for the increase in RWC given that airspaces in the mesophyll are the sites of highest resistance to water flow in leaves [37,38,39]. The increase in intercellular spaces, associated with the increased RWC, may have significantly reduced water losses and improved the photosynthetic efficiency of irradiated seeds under FS and RB light regimes. IR generally impairs photosynthesis and stomatal conductance, irrespective of the kind of radiation and dose [40,41,42,43,44]. Consistent with other studies, the photosynthetic metabolism of Microtom was affected by ionizing radiation.

Moreover, the most novel result is the reversal of the effect of ionizing radiation when plants are grown under different light quality regimes. Under FL regime, irradiated plants decreased photosynthesis compared to the control; conversely, an opposite trend was observed under the RB regime. The higher photosynthetic rates can be ascribed to the high stomatal conductance of RB compared to FL plants, due to the stimulation of blue wavelengths on stomatal opening [45,46,47,48,49]. Furthermore, the increased incidence of intercellular spaces (IS%) within the mesophyll may have also favored the CO_2_ diffusion to carboxylation sites, increasing the photosynthetic rate and Rubisco activity. Consistent with high photosynthetic rates, FS and RB irradiated plants exhibited a greater amount of Rubisco than controls. It has been demonstrated that the higher quantity of blue wavelengths in FS and RB regimes elicits a positive effect on Rubisco expression and plant photosynthetic capacity [4,50]. Irrespective of the LQ regime, total proteins did not vary among control plants but changed among irradiated ones. The lowest value was found in IR-FL plants perhaps because IR may have induced the proteins’ degradation or inhibited their synthesis [51,52]. Conversely, IR-FS and IR-RB plants showed a greater protein content likely due to the positive effect exerted by blue and red wavelengths on the protein synthesis [32,53,54,55]. It is also reasonable to assume that the increase in Rubisco amount significantly contributed to the total protein rise under these treatments.

The interaction IR × LQ also significantly affected the leaf antioxidant properties. As expected, the highest portion of blue light in the RB regime determined the increase in the total antioxidant capacity, flavonoids and polyphenols [32,36,56], but in combination with ionizing radiation, it induced a reduction in antioxidants. An increase in polyphenols and flavonoids in irradiated plants is expected to counteract the radio-induced oxidative damages [7,21,26,57,58]. In our case, the decrease in antioxidants in irradiated plants may indicate that the dose of Ca 25 Gy is not dangerous for plants.

‘Microtom’ plants showed no detrimental effect at the dose of IR used in this study. The heatmap clearly separated IR-RB plants from the others, evidencing that if irradiated plants are grown under specific light regimes, such as RB, a beneficial effect in terms of gas exchanges can be obtained. Moreover, the interplay between LQ and IR significantly modulates the tomato plant’s morphological responses, further affecting the intrinsic dwarf habitus of this cultivar. Besides high photosynthetic gain, the dwarf growth is one of the most desirable requirements for cultivation in a high plant density condition or slim volumes, such as those available in Space systems [15,26,59,60]. The plant bioactive compounds such as antioxidants, polyphenols and flavonoids decreased in irradiated ‘Microtom’ plants compared to control, especially in combination with RB growth regime, indicating that these plants do not perceive the dose of ionizing radiation used in our study as a potential stress.

## 4. Materials and Methods

### 4.1. Plant Material, Sample Irradiation and Experimental Plan

Dry seeds of *Solanum lycopersicum* L. cv ‘Microtom’, provided by Holland Online Vof (Amsterdam, The Netherlands), from the same production collection, were divided into two cohorts: “non-irradiated control (C)” and “irradiated (IR)” group. For the latter, seeds were treated with Ca heavy ions [isotope ^48^Ca; E: 200 MeV·u^−1^ (monoenergetic), LET: 180 keV·μm^−1^; dose rate 1 Gy·min^−1^] at a dose of 25 Gy. The sample irradiation was executed at GSI Helmholtzzentrum für Schwerionenforschung (Darmstadt, Germany), using a pencil beam in a spread-out Bragg peak (SOBP), in the heavy-ion synchrotron (SIS). The dose of 25 Gy, under the threshold for occurrence of DNA damage [61], was chosen to not prevent the plant development but rather to obtain a stimulatory effect on plant growth and physiology. Non-irradiated seeds served as controls.

**Figure 7:**
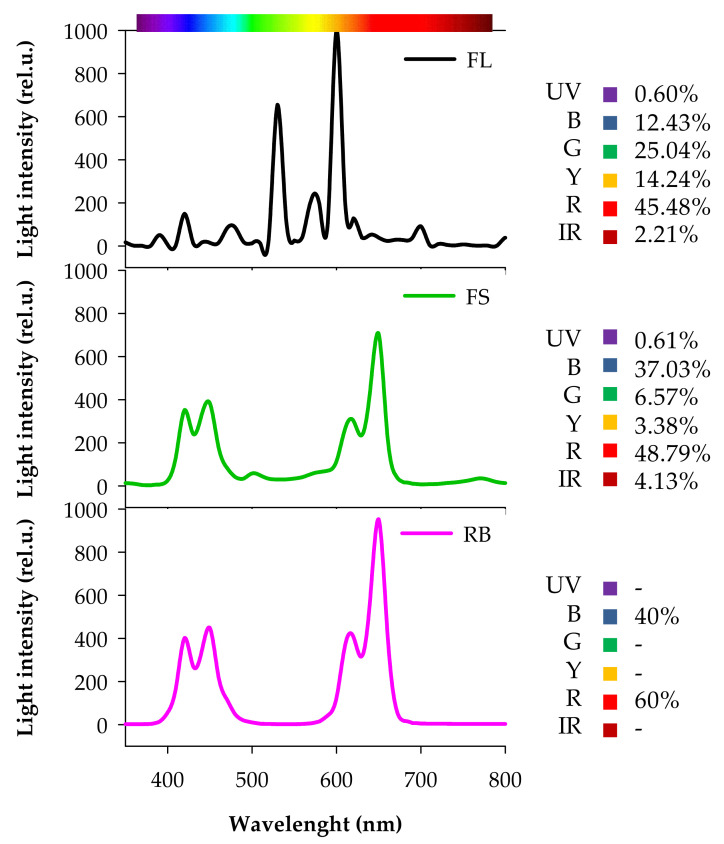
Composition of the light spectra used in the experiment. FL, white fluorescent tubes; FS, full-spectrum, LED; RB, red-blue, LED. Photon flux density: 360 µmol m^−2^s^−1^. Irradiance Range: 350–800 nm. UV, ultra-violet (350–390 nm); B, Blue (390–500 nm); G, Green (500–560 nm); Y, Yellow (560–600 nm); R, Red (600–700 nm); IR, Far-Red (700–800 nm).

C and IR seeds were sown in 1.2 L pots filled with soil (86% peat, 9% sand, 3% quartz sand, 2% perlite) and kept in the dark until germination. After germination (15 days after sowing, DAS), seedlings were transferred in a growth chamber under three different light quality regimes (5 pots per each light treatment): white fluorescent (FL), full-spectrum (FS), and red-blue (RB). FL was provided by a combination of fluorescent tubes (Lumilux L36W/640 and L36W/830, Osram, München, Germany), the FS and RB regimes were obtained by an arrangement of light emitting diodes (LEDs) (LedMarket Ltd., Plovdiv, Bulgaria). More specifically, FS was composed by an assortment of far-red, red, yellow, green, blue, UV-A and white light, while RB derived from a mix of red (R: 60%) and blue B: 40%) light. The spectral composition of the light regimes was assessed through a SR-3000A spectro-radiometer at a resolution of 10 nm (Macam Photometrics Ltd., Livingston, Scotland, UK) (Figure 7).

The photosynthetic photon flux density (PPFD) in the growth chamber was fixed at 360 μmol photons m^−2^ s^−1^ at the top of the canopy for all LQ regimes with photoperiod of 14 h. The relative air humidity was 60–70% and day/night air temperature 25/20 °C ± 2 °C. Plants were irrigated weekly to field capacity to reintegrate water lost by evapotranspiration and fertilized weekly with half-strength Hoagland’s solution.

Plant growth was followed until 120 DAS up to fruit ripening. The seed germination (15 DAS), the beginning of flowering (60 DAS) and the achievement of the maximum stem elongation (70 DAS) were used as reference to normalize the plant growth because all individuals, regardless of the different treatments, reached full germination at 15 DAS, maximum stem elongation at 70 DAS, and started flowering at 60 DAS.

The morphological, anatomical and physiological measurements were conducted at 70 DAS; flower and fruit number were monitored from their first appearance up to 120 DAS, considering for each plant the sum of flowers and fruits produced and harvested.

We used the same composite leaf (the fourth leaf from the stem base) for all determinations. In particular, the central and lateral leaflets of the fourth leaf were used for gas exchange measurements, while the central leaflet was sampled for total protein and Rubisco content analyses. In contrast, the lateral leaflets were used to determine leaf anatomy and biochemical parameters, respectively.

All determinations were carried out on biological replicates.

### 4.2. Seed Germination, Biometrical Measurement and Leaf Functional Traits

The germination test was performed on 10 seeds per treatment and repeated three times for a total of 30 control seeds and 30 irradiated seeds. The germination percentage (G%) was estimated as

G% = (Number of germinated seeds/Total number of seeds) × 100(1)

The effect of irradiation and light quality on biometric characteristics (plant height, total plant leaf area, leaf, flower, and fruit number) was evaluated on five plants per treatment. The leaf functional traits (leaf area, LA; specific leaf area, SLA; leaf dry matter content, LDMC; relative water content, RWC) were estimated according to Cornelissen et al. [62]. Leaf area was determined by the Image J software (Image Analysis Software, Rasband, NIH, Bethesda, MD, USA) and used to calculate SLA (cm^2^ g^−1^) as leaf area to leaf dry mass ratio. LDMC (g g^−1^) was estimated as leaf dry mass to saturated fresh mass; RWC was calculated as (leaf fresh mass − leaf dry mass)/(leaf saturated fresh mass − leaf dry mass). The saturated fresh mass was achieved by submerging the leaf petiole in distilled water for 48 h in the dark at 5 °C, whereas the dry mass was obtained after oven-drying leaves at 75 °C for 48 h.

To quantify the total plant leaf area, the image of each single leaf was acquired by a digital camera. The leaf lamina expansion was measured using Image J software (Image Analysis Software, Rasband, NIH, Bethesda, MD, USA). The plant leaf area was calculated as the sum of all leaf areas of each plant and expressed as cm^2^.

### 4.3. Anatomical Analyses

The leaf anatomical analyses were carried out at 70 DAS on three fully expanded mature leaves collected from three different plants per treatment. Briefly, segments from the middle leaflets of compound leaves were cut and quickly immersed in the fixative solution FAA (40% formaldehyde/glacial acetic acid/50% ethanol, 5/5/90 *v*/*v*/*v*). Subsamples (5 mm^2^) were dissected from the leaflet lamina, flattened on a microscope slide, mounted with water and observed under a transmitting light microscope (BX51; Olympus, Germany) to determine the stomatal density (n mm^−2^). Leaf lamina thickness, palisade and spongy parenchyma thickness were determined on another group of subsamples (5 mm^2^) which were dehydrated in ethanol series (50%, 70%, 95% ethanol) and embedded in the acrylic resin JB-4 (Sigma Aldrich, St. Louis, MO, USA). Cross-sections of 5 μm thickness were achieved by a rotatory microtome and stained with 0.5% toluidine blue in water [63]. The sections were observed under a transmitted light microscope (BX51; Olympus) and images were collected by a digital camera (EP50; Olympus) and analyzed with the software Olympus CellSens 2.3.

The thickness of leaf lamina and palisade and spongy parenchyma was measured in three points along the lamina, eluding veins. The occurrence of intercellular spaces in the spongy parenchyma was measured as the percentage of tissue occupied by intercellular spaces over a fixed surface along the mesophyll.

### 4.4. Gas Exchange Measurements

Leaf gas-exchange measurements were performed at 70 DAS by means of a portable leaf gas-exchange system (LCpro+, ADC BioScientific, UK) on ten fully expanded leaflets per treatment (five plants, two leaflets per plant). The apical leaflet was clamped into cuvette, and measurements were carried out at leaf temperature of 25 ± 2 °C, relative humidity of 50–60% and ambient CO_2_ (400 μmol mol^−1^). Light response curves (LRC) were performed by exposing leaflets to white light ranging from 70 to 1200 μmol m^−2^ s^−1^ PPFD. The net CO_2_ assimilation (*P*_N_), stomatal conductance (*g*_sH2O_) and intracellular CO_2_ concentration (*C*_i_) were calculated by the software operating in the gas-exchange system following the equations of von Caemmerer and Farquhar [64]. In LRC, gas-exchange parameters were recorded after the achievement of the steady state (about 5–10 min) for each PPFD step. Parameters derived by light response curves (*P*_Nsat_, *I*_sat_, and Φ_CO2_) were calculated following Abe et al. [65].

### 4.5. Total Protein and Rubisco Amount

The protein extraction and Rubisco amount were determined on five leaves (one leaf per plant) per treatment. We sampled for the analyses always the fourth leaf from the stem base to avoid differences due to diverse leaf age or light exposition might compromise the results of these determinations.

The protein extraction was carried out on 0.3 g of fresh material for each sample according to method reported in Wang et al. [66]. The extracts were quantified by the Bradford assay [67] (BioRad Protein Assay Dye Reagent Concentrate; Bio-Rad Laboratories, Milan, Italy) determining the absorbance at 595 nm by a spectrophotometer (UV-VIS Cary 100; Agilent Technologies, Palo Alto, CA, USA). The bovine serum albumin (BSA) was used as standard. SDS-PAGE (10%) was performed following the procedure of Arena et al. (2019), using Pro-liner 3-colour (Cyanagen Srl, Bologna, Italy) as marker, and Laemmli loading buffer to track the separation of proteins.

Western blot analysis was achieved using a blocking solution (100 mM Tris-HCl pH 8.0, 150 mM NaCl, 0.1% Tween 20, 2.5% BSA) and primary antibodies (Agrisera, Vännäs, Sweden) to reveal Rubisco (anti-RbcL, rabbit polyclonal serum, 1:10,000 *v*/*v*), and Actin protein (anti-ACT, mouse monoclonal, 1:1000 *v*/*v*) utilized as a loading control. Anti-Rabbit IgG (H&L), HRP conjugated (1:6000 *v*/*v*) was used as secondary antibody for Rubisco, while Rabbit-Anti-Mouse IgG (H&L), HRP conjugated (1:2000 *v*/*v*) was used for Actin. The immunorevelation was carried out with the kit for chemiluminescence (Westar supernova, Cyanagen Srl, Bologna, Italy) via ChemiDoc System (Bio-Rad). The software Image Lab (Bio-Rad Laboratories, Hercules, CA, USA) was utilized for the densitometric analysis to obtain quantitative information associated with the individual bands.

Each Rubisco band was normalized to the corresponding actin band. Density values were expressed in arbitrary units and represented as bar diagrams showing pixel volumes of protein bands.

### 4.6. Leaf Biochemical Analyses

The antioxidant capacity, total polyphenols and flavonoids were determined on five fully expanded leaves (one leaf per plant) per treatment.

The antioxidant capacity was determined with the ferric reducing antioxidant power (FRAP) assay following the procedure described in George et al. [68] and modified by Vitale et al. [69]. An amount of 0.250 g of samples was grounded in liquid nitrogen, mixed with 60:40 (*v*/*v*) methanol/water solution, and centrifuged for 15 min at 14.000 rpm at 4 °C. At each extract, the FRAP reagents composed by 300 mM acetate buffer pH 3.6; 10 mM tripyridyltriazine (TPTZ), 40 mM HCl and 12 mM FeCl_3_, were added in a ratio 16.6:1.6:1.6 (*v*/*v*), respectively. Subsequently, the extracts were darkened for 1 h, and the absorbance was read at 593 nm through a spectrophotometer (UV-VIS Cary 100, Agilent Technologies, Palo Alto, CA, USA). The total antioxidant capacity was quantified as µmol Trolox (6-hydroxy-2,5,7,8-tetramethylchroman-2-carboxylic acid) equivalents per gram of fresh weight (µmol TE g^−1^ FW) using the Trolox as standard.

The total polyphenol content was assessed following the procedure reported in Arena et al. [15]. Briefly, 0.02 g of powdered samples were extracted in methanol at 4 °C and centrifuged for 5 min at 11.000 rpm. Extracts were mixed with 1:1 (*v*/*v*) 10% Folin–Ciocâlteu reagent and 5:1 (*v*/*v*) 700 mM Na_2_CO_3_ solution after 3 min. The absorbance of samples was measured at 765 nm with a spectrophotometer (UV-VIS Cary 100, Agilent Technologies, Palo Alto, CA, USA) after 2 h of incubation in the darkness. The total polyphenol content was expressed as mg of gallic acid equivalents per gram of fresh weight (mg GAE g^−1^ FW) by a calibration curve built with gallic acid as standard.

Total flavonoid content was evaluated according to Moulehi et al. [70] and Sun et al. [71]. An amount of 0.02 g of powered sample, diluted with 80% methanol aqueous solution, was mixed with 3:1 (*v*/*v*) sodium nitrite (NaNO_2_) at 5%. Following 6 min, 10% AlCl_3_ (aluminium chloride) and NaOH (1 M) were added to the mixture adjusting the volume with distilled water. The absorbance of samples was read at 510 nm. Total flavonoid content was calculated by means of a catechin standard curve and expressed as mg catechin equivalent per gram of fresh weight (mg CE g^−1^ FW).

### 4.7. Statistical Analysis

Statistical analysis was performed with the SigmaPlot 12 software (Jandel Scientific, San Rafael, CA, USA). The outcomes of IR on germination were evaluated by *t*-test considering a significance level of *p* < 0.05. The influence of the two different independent factors, i.e., ionizing radiation (IR) and light quality treatments (LQ), and their possible interaction on biometrical, anatomical and functional parameters were assessed by two-way ANOVA. The normality was checked by the Kolmogorov–Smirnov test. The Student–Newman–Keuls (SNK) test was applied for all pairwise multiple comparison tests with a significance level of *p* < 0.05. When the interaction between IR and LQ was significant, data were further analyzed by one-way ANOVA, and multiple comparison tests were done with SNK coefficient.

All parameters were visualized by a heatmap (heatmap function). The heatmap was built using the ClustVis program package (https://biit.cs.ut.ee/clustvis/online (accessed on 23 July 2021)) and grouping both rows and columns with Euclidean distance and average linkage. The numeric differences in the heatmap are highlighted by a color scale where the red and blue colors indicate the increasing and the decreasing of each parameters, respectively.

## 5. Conclusions

This study demonstrated that different light spectral composition modifies the morphological and physiological attributes of ‘Microtom’ plants sprouted from seeds irradiated with Ca-ions at 25 Gy, inducing dwarf growth and ameliorating the plant water relationships. The completion of the life cycle was observed in all plants, irrespective of light regimes. In particular, the RB treatment enhanced the compact architecture in irradiated plants, representing a valuable trait for the limited volumes at disposal for plant cultivation in Space. The RB light growth regime also improved the photosynthetic performance of irradiated plants by modulating stomatal conductance and Rubisco content exerted by blue light. At the anatomical level, the occurrence of more intercellular spaces in RB irradiated plants likely improved the mesophyll conductance and induced an increase in Rubisco expression. This study suggested that specific LQ regimes modify functional attributes in ‘Microtom’ irradiated plants, favouring photosynthesis. This result is particularly encouraging in ‘Microtom’ cultivation on board of Controlled Ecological Life Support Systems (CELSS) as a food supplement for astronaut diet in long-term Space missions.

## Figures and Tables

**Table 1:** Analysis of variance and comparison of means for morphological traits of ‘Microtom’ plants in response to ionizing radiation (IR), light quality (LQ), and their interaction (IR × LQ). C: mean of non-irradiated control plants (C-FL; C-FS; C-RB); IR: mean of irradiated plants (IR-FL; IR-FS; IR-RB). FL: mean of C-FL and IR-FL plants; FS: mean of C-FS and IR-FS plants; RB: mean of C-RB and IR-RB plants. Different letters within each column indicate significant differences according to Student–Newman–Keuls multiple comparison test (*p* < 0.05). NS: not significant; * *p* < 0.05; ** *p* < 0.01; *** *p* < 0.001.

	Height	TLA	Leaves	Flowers	Fruits
**IR**					
C	7.1 a	304 a	10 b	57 a	24 a
IR	5.5 b	271 b	11 a	51 b	23 a
**LQ**					
FL	7.7 a	323 a	11 a	50 b	25 a
FS	5.7 b	276 b	11 a	50 b	26 a
RB	5.4 b	263 b	11 a	62 a	20 b
**Significance**					
IR	***	***	*	*	NS
LQ	***	***	NS	***	**
IR × LQ	*	*	***	***	NS

Height: plant height (cm); TLA: total plant leaf area (cm^2^); Leaves: number of leaves per plant (n plant^−1^); Flowers: number of flowers per plant (n plant^−1^); Fruits: number of fruits per plants (n plant^−1^).

**Table 2:** Analysis of variance and comparison of means for functional and anatomical traits of ‘Microtom’ plants in response to ionizing radiation (IR), light quality (LQ), and their interaction (IR × LQ). C: mean of non-irradiated control plants (C-FL; C-FS; C-RB); IR: mean of irradiated plants (IR-FL; IR-FS; IR-RB). FL: mean of C-FL and IR-FL plants; FS: mean of C-FS and IR-FS plants; RB: mean of C-RB and IR-RB plants. Different letters within each column indicate significant differences according to Student–Newman–Keuls multiple comparison test (*p* < 0.05). NS-not significant; * *p* < 0.05; ** *p* < 0.01; *** *p* < 0.001.

	Functional Traits				Anatomical Traits				
	LA	SLA	LDMC	RWC	LT	PT	ST	IS	SD
**IR**									
C	9.4 b	210 a	0.11 b	60 b	231 a	99 a	100 a	31 a	215 a
IR	13 a	219 a	0.12 a	66 a	228 a	94 a	97 a	33 a	221 a
**LQ**									
FL	13 a	212 a	0.12 a	54 b	188 c	74 c	81 c	24 c	213 a
FS	10 b	217 a	0.11 a	66 a	221 b	92 b	94 b	32 b	222 a
RB	9.8 b	214 a	0.11 a	69 a	280 a	123 a	120 a	40 a	217 a
**Condition**									
C-FL	9.6 b	219 a	0.11 a	55 d	200 bc	80 bc	86 bc	28 b	181 a
IR-FL	17 a	205 a	0.12 a	53 d	175 c	68 c	76 c	21 c	246 a
C-FS	9.1 b	203 a	0.11 a	62 c	214 b	92 b	92 b	30 b	234 a
IR-FS	12 b	231 a	0.11 a	69 b	228 b	93 b	96 b	33 b	210 a
C-RB	9.5 b	207 a	0.11 a	62 c	280 a	125 a	120 a	36 b	229 a
IR-RB	10 b	222 a	0.12 a	75 a	280 a	121 a	119 a	48 a	206 a
**Significance**									
IR	***	NS	*	**	NS	NS	NS	NS	NS
LQ	***	NS	NS	NS	***	***	***	***	NS
IR × LQ	***	NS	NS	*	NS	NS	NS	*	NS

LA: Leaf area (cm^2^); SLA: Specific leaf area (cm^2^ g^−1^); LDMC: Leaf dry matter content (g g^−1^); RWC: Relative water content (%); LT: lamina thickness (μm); PT: palisade thickness (μm); ST: spongy thickness (μm); IS: intercellular spaces (%); SD: Stomata density (n mm^−2^).

**Table 3:** Analysis of variance and comparison of means for photosynthetic functional traits of ‘Microtom’ plants in response to ionizing radiation (IR), light quality (LQ), and their interaction (IR × LQ). C: mean of non-irradiated control plants (C-FL; C-FS; C-RB); IR: mean of irradiated plants (IR-FL; IR-FS; IR-RB). FL: mean of C-FL and IR-FL plants; FS: mean of C-FS and IR-FS plants; RB: mean of C-RB and IR-RB plants. Different letters in each column indicate significant differences according to Student–Newman–Keuls test (*p* < 0.05). NS-not significant; * *p* < 0.05; ** *p* < 0.01; *** *p* < 0.001.

	*P*_Nsat_	*I*_sat_	Φ_CO2_
**IR**			
C	23 a	508 a	0.047 a
IR	25 a	518 a	0.050 a
**LQ**			
FL	23 b	525 a	0.044 b
FS	26 a	517 a	0.051 a
RB	25 a	497 a	0.052 a
**Condition**			
C-FL	25 b	558 a	0.045 b
IR-FL	20 c	492 a	0.042 b
C-FS	24 b	509 a	0.048 a
IR-FS	27 a	524 a	0.053 a
C-RB	22 c	457 a	0.049 a
IR-RB	29 a	537 a	0.055 a
**Significance**			
IR	NS	NS	NS
LQ	*	NS	**
IR × LQ	***	NS	*

P_Nsat_: light saturated CO_2_ uptake (μmol CO_2_ m^−2^s^−1^); I_sat_: light saturation point (μmol photons m^−2^s^−1^); Φ_CO2_: quantum yield of photosynthesis (μmol CO_2_ mmol^−1^ photons).

**Table 4:** Analysis of variance and means comparison for biochemical traits of ‘Microtom’ plants in response to ionizing radiation (IR), light quality (LQ), and their interaction (IR × LQ). C: mean of non-irradiated control plants (C-FL; C-FS; C-RB); IR: mean of irradiated plants (IR-FL; IR-FS; IR-RB). FL: mean of C-FL and IR-FL plants; FS: mean of C-FS and IR-FS plants; RB: mean of C-RB and IR-RB plants. Different letters in each column indicate significant differences according to Student–Newman–Keuls test (*p* < 0.05). NS-not significant; * *p* < 0.05; ** *p* < 0.01; *** *p* < 0.001.

	TAC	TPC	FLAV	PROT	RUB
**IR**					
C	2.9 a	0.92 a	20 a	0.30 b	0.90 b
IR	2.8 a	0.83 b	18 b	0.41 a	1.12 a
**LQ**					
FL	2.6 b	0.83 b	18 b	0.27 c	0.92 b
FS	3.0 a	0.87 b	19 b	0.34 b	0.81 b
RB	2.9 a	0.92 a	20 a	0.45 a	1.30 a
**Significance**					
IR	NS	***	***	***	*
LQ	***	**	**	***	**
IR × LQ	***	NS	NS	***	*

TAC: total antioxidant capacity (μmol TE g^−1^ FW); TPC: total polyphenol content (mg GAE g^−1^ FW); FLAV: total flavonoids (mg CE g^−1^ FW); PROT: total proteins (μg BSA eq mg^−1^ FW); RUB: Rubisco amount (arbitrary units).

## Data Availability

The data supporting the results of this study are accessible from the corresponding author (CA), upon reasonable request.

## References

[1] Singh D., Basu C., Meinhardt-Wollweber M., Roth B. (2015). LEDs for energy efficient greenhouse lighting. Renew. Sust. Energ. Rev..

[2] Bantis F., Smirnakou S., Ouzounis T., Koukounaras A., Ntagkas N., Radoglou K. (2018). Current status and recent achievements in the field of horticulture with the use of light-emitting diodes (LEDs). Sci. Hortic..

[3] Morrow R.C. (2008). LED lighting in horticulture. HortScience.

[4] Izzo L.G., Hay Mele B., Vitale L., Vitale E., Arena C. (2020). The role of monochromatic red and blue light in tomato early photomorphogenesis and photosynthetic traits. Environ. Exp. Bot..

[5] Paradiso R., Proietti S. (2021). Light-Quality Manipulation to Control Plant Growth and Photomorphogenesis in Greenhouse Horticulture: The State of the Art and the Opportunities of Modern LED Systems. J. Plant Growth Regul..

[6] Yavari N., Tripathi R., Wu B.S., MacPherson S., Singh J., Lefsrud M. (2021). The effect of light quality on plant physiology, photosynthetic, and stress response in Arabidopsis thaliana leaves. PLoS ONE.

[7] Arena C., De Micco V., De Maio A. (2014). Growth alteration and leaf biochemical responses in *P. vulgaris* plants exposed to different doses of ionizing radiation. Plant Biol..

[8] Honda I., Kikuchi K., Matsuo S., Fukuda M., Saito H., Ryuto H., Fukunishi N., Abe T. (2006). Heavy-ioninduced mutants in sweet pepper isolated by M1 plant selection. Euphytica.

[9] Hou S., Sun L., Zhang Y., Wu D., Guan L., Gao Q., Li W., Dang B., Xie H., Zhou L. (2008). Mutagenic effects of *Brassica napus* by ^12^C^6+^ ion beam. Nucl. Technol..

[10] Xie Z.K., Wang Y.J., Xie H.M., Guo Z.H., Wei Z.Q. (2008). Study of mutation breeding with heavy ion irradiation on potatoes. Nucl. Phys. Rev..

[11] Kharkwal M.C., Shu Q.Y., Foster B.P., Nakagawa H. (2012). A brief history of plant mutagenesis. Plant Mutation Breeding and Biotechnology.

[12] Dong X., Yan X., Li W. (2016). Plant mutation breeding with heavy ion irradiation at IMP. J. Agric. Sci..

[13] Jo Y.D., Kim S.H., Hwang J.E., Kim Y.S., Kang H.S., Kim S.W., Kwon S.J., Ryu J., Kim J.B., Kang S.Y. (2016). Construction of mutation populations by Gamma-ray and Carbon beam irradiated in Chili pepper (*Capsicum annuum* L.). Hortic. Environ. Biotechnol..

[14] Oladosu Y., Rafii M.Y., Abdullah N., Hussin G., Ramli A., Rahim H.A., Miah G., Usman M. (2016). Principle and application of plant mutagenesis in crop improvement: A review. Biotechnol. Biotechnol. Equip..

[15] Arena C., Vitale E., Hay Mele B., Cataletto P.R., Turano M., Simoniello P., De Micco V. (2019). Suitability of Solanum lycopersicum L. ‘Microtom’ for growth in Bioregenerative Life Support Systems: Exploring the effect of high-LET ionising radiation on photosynthesis, leaf structure and fruit traits. Plant Biol..

[16] De Micco V., Arena C., Pignalosa D., Durante M. (2011). Effects of sparsely and densely ionizing radiation on plants. Radiat. Environ. Biophys..

[17] Wei Z., Liu Y., Wang G., Chen X., Li H., Yang H., Wang L., Gao Q., Wang C., Wang Y. (1995). Biological effects of carbon ions with medium energy on plant seeds. Radiat. Res..

[18] Hase Y., Shimono K., Inoue M., Tanaka A., Watanabe H. (1999). Biological effects of ion beams in *Nicotiana tabacum* L. Radiat. Environ. Biophys..

[19] Komai F., Shikazono N., Tanaka A. (2003). Sexual modification of female spinach seeds (*Spinacia oleracea* L.) by irradiation with ion particles. Plant Cell Rep..

[20] Sjahril R., Riadi M., Raffiundin S.T., Toriyama K., Abe T., Trisnawaty A.R. (2018). Effect of heavy ion beam irradiation on germination of local Toraja rice seed (M1-M2) mutant generation. IOP Conf. Ser. Earth Environ. Sci..

[21] Arena C., De Micco V., Macaeva E., Quintens R. (2014). Space radiation effects on plant and mammalian cells. Acta Astronaut.

[22] Manchester L.C., Tan D.X., Reiter R.J., Park W., Monis K., Qi W. (2000). High levels of melatonin in the seeds of edible plants: Possible function in germ tissue protection. Life Sci..

[23] Okazaki M., Ezura H. (2009). Profiling of melatonin in the model tomato (*Solanum lycopersicum* L.) cultivat Micro-Tom. J. Pineal Res..

[24] Stürtz M., Cerezo A.B., Cantos-Villar E., Garcia-Parrilla M.C. (2011). Determination of the melatonin content of different varieties of tomatoes (*Lycopersicon esculentum*) and strawberries (*Fragaria ananassa*). Food Chem..

[25] Mei M., Deng H., Lu Y., Zhuang C., Liu Z., Qiu Y., Yang T.C. (1994). Mutagenic effects of heavy ion radiation in plants. Adv. Space Res..

[26] De Micco V., Paradiso R., Aronne G., De Pascale S., Quarto M., Arena C. (2014). Leaf anatomy and photochemical behaviour of *Solanum lycopersicum* L. plants from seeds irradiated with low-LET ionizing radiation. Sci. World J..

[27] Nechitailo G.S., Jinying L., Huai X., Yi P., Chongqin T., Min L. (2005). Influence of long-term exposure to space flight on tomato seeds. Adv. Space Res..

[28] Dougher T.A., Bugbee B. (2004). Long-term blue effect on histology of lettuce and soybean leaves and stems. J. Am. Soc. Hortic. Sci..

[29] Nanya K., Ishigami Y., Hikosaka S., Goto E. (2012). Effects of blue and red light on stem elongation and flowering of tomato seedlings. Acta Hortic..

[30] Xiaoying L., Shirong G., Taotao C., Zhigang X., Tezuka T. (2012). Regulation of the growth and photosynthesis of cherry tomato seedlings by different light irradiations of light emitting diodes (LED). Afr. J. Biotechnol..

[31] Arena C., Tsonev T., Doneva D., De Micco V., Michelozzi M., Brunetti C., Centritto M., Fineschi S., Velikova V., Loreto F. (2016). The effect of light quality on growth, photosynthesis, leaf anatomy and volatile isoprenoids of a monoterpene-emitting herbaceous species (*Solanum lycopersicum* L.) and an isoprene-emitting tree (*Platanus orientalis* L.). Environ. Exp. Bot..

[32] Vitale E., Velikova V., Tsonev T., Ferrandino I., Capriello T., Arena C. (2021). The interplay between light quality and biostimulant application affects the antioxidant capacity and photosynthetic traits of soybean (*Glycine max*. L. Merrill). Plants.

[33] Poorter L., Bongers F. (2006). Leaf traits are good predictors of plant performance across 53 rain forest species. Ecology.

[34] Ryser P., Urbas P. (2000). Ecological significance of leaf life span among Central European grass species. Oikos.

[35] Yorio N.C., Goins G.D., Kagie H.R., Wheeler R.M., Sager J.C. (2001). Improving Spinach, Radish, and Lettuce Growth under Red Light emitting Diodes (LEDs) with Blue Light Supplementation. Hortic. Sci..

[36] Agarwal A., Dutta Gupta S. (2016). Impact of light-emitting diodes (LEDs) and its potential on plant growth and development in controlled-environment plant production system. Curr. Biotechnol..

[37] Buckley T.N., John G.P., Scoffoni C., Sack L. (2015). How does leaf anatomy influence water transport outside the xylem?. Plant Physiol..

[38] Amitrano C., Arena C., Rouphael Y., De Pascale S., De Micco V. (2019). Vapour pressure deficit: The hidden driver behind plant morphofunctional traits in controlled environments. Ann. Appl. Biol..

[39] Amitrano C., Arena C., Cirillo V., De Pascale S., De Micco V. (2021). Leaf morpho-anatomical traits in *Vigna radiata* L. affect plant photosynthetic acclimation to changing vapor pressure deficit. Environ. Exp. Bot..

[40] Thiede M.E., Link S.O., Fellows R.J., Beedlow P.A. (1995). Effect of gamma radiation on stem diameter growth, carbon gain and biomass partitioning in Helianthus annus. Environ. Exp. Bot..

[41] Ursino D.J., Schefski H., McCabe J. (1977). Radiation-induced changes in rate of photosynthetic CO_2_ uptake in soybean plants. Environ. Exp. Bot..

[42] Jia C.F., Li A.L. (2008). Effect of gamma radiation on mutant induction of *Fagopyrum dibotrys* Hara. Photosynthetica.

[43] Moghaddam S.S., Jaafar H., Ibrahim R., Rahmat A., Maheran A.A., Philip E. (2011). Effects of Acute Gamma Irradiation on Physiological Traits and Flavonoid accumulation of Centella asiatica. Molecules.

[44] Fan J., Shi M., Huang J.Z., Xu J., Wang Z.D., Guo D.P. (2014). Regulation of photosynthetic performance and antioxidant capacity by ^60^Co γ-irradiation in *Zizania latifolia* plants. J. Environ. Radioact..

[45] Akoyunoglou G., Anni H., Senger H. (1984). Blue light effect on chloroplast development in higher plants. Blue Light Effects in Biological Systems.

[46] Sæbø A., Krekling T., Applegren M. (1995). Light quality affects photosynthesis and leaf anatomy of birch plantlets in vitro. Plant Cell Tissue Organ Cult..

[47] Brown C.S., Schuerger A.C., Sager J.C. (1995). Growth and photomorphogenesis of pepper plants under red light-emitting diodes with supplemental blue or far-red lighting. J. Am. Soc. Hortic. Sci..

[48] Goins G.D., Yorio N.C., Sanwo M.M., Brown C.S. (1997). Photomorphogenesis, photosynthesis, and seed yield of wheat plants grown under red light-emitting diodes (LED) with and without supplemental blue light. J. Exp. Bot..

[49] Inoue S.I., Kinoshita T. (2017). Blue light regulation of stomatal opening and the plasma membrane H^+^-ATPase. Plant Physiol..

[50] Wang H., Gu M., Cui J., Shi K., Zhou Y., Yu J. (2009). Effects of light quality on CO_2_ assimilation, chlorophyll-fluorescence quenching, expression of Calvin cycle genes and carbohydrate accumulation in *Cucumis sativus*. J. Photochem. Photobiol. B.

[51] Hammeed A., Shah T.M., Atta B.M., Haq M.A., Sayed H. (2008). Gamma Irradiation effects on seed germination and growth, protein content, peroxidase and protease activity, lipid peroxidation in Desi and Kabuli Chickpea. Pak. J. Bot..

[52] Kiong A.L.P., Lai A.G., Hussein S., Harun A.R. (2008). Physiological Responses of *Orthosiphon stamineus* Plantles to Gamma Irradiation. Am. Eurasian J. Sustain. Agric..

[53] Zhang L., Liu S., Zhang Z., Yang R., Yang X. (2010). Dynamic effects of different light qualities on pea sprouts quality. North. Hortic..

[54] Li H., Tang C., Xu Z., Liu X., Han X. (2012). Effects of Different Light Sources on the Growth of Non-heading Chinese Cabbage (*Brassica campestris* L.). J. Agric. Sci..

[55] Amitrano C., Vitale E., De Micco V., Arena C. (2018). Light fertilization affects growth and photosynthesis in mung bean (*Vigna radiata*) plants. J. Environ. Account. Manag..

[56] Bian Z.H., Yang Q.C., Liu W.K. (2015). Effects of light quality on the accumulation of phytochemicals in vegetables produced in controlled environments: A review. J. Sci. Food Agric..

[57] Graham L.E., Kodner R.B., Fisher M.M., Graham J.M., Wilcox L.W., Hackney J.M., Obst J., Bilkey P.C., Hanson D.T., Cook M.E., Hemsley A.R., Poole I. (2004). Early land plant adaptations to terrestrial stress: A focus on phenolics. The Evolution of Plant Physiology.

[58] Zhang R., Kang K.A., Kang S.S., Park J.W. (2011). Morin (2′,3,4′,5,7-pentahydroxyflavone) protected cells against γ radiation-induced oxidative stress. Basic Clin. Pharmacol. Toxicol..

[59] Colla G., Rouphael Y., Cardarelli M., Mazzucato A., Olimpieri I. (2007). Growth, yield and reproduction of dwarf tomato grown under simulated microgravity conditions. Plant Biosyst..

[60] Saito T., Ariizumi T., Okabe Y., Asamizu E., Hiwasa-Tanase K., Fukuda N., Mizoguchi T., Yamazaki Y., Aoki K., Ezura H. (2011). TOMATOMA: A novel tomato mutant database distributing Micro-Tom mutant collections. Plant Cell Physiol..

[61] Kazama Y., Hirano T., Saito H., Liu Y., Ohbu S., Hayashi Y., Abe T. (2011). Characterization of highly efficient heavy-ion mutagenesis in Arabidopsis thaliana. BMC Plant Biol..

[62] Cornelissen J.H.C., Lavorel S., Garnier E., Diaz S., Buchmann N., Gurvich D.E., Reich P.B., Ter Steege H., Morgan H.D., Van Der Heijden M.G.A. (2003). A handbook of protocols for standardised and easy measurement of plant functional traits worldwide. Aust. J. Bot..

[63] Feder N., O’brien T. (1968). Plant microtechnique: Some principles and new methods. Am. J. Bot..

[64] Von Caemmerer S., Farquhar G.D. (1981). Some relationship between the biochemistry of photosynthesis and the gas exchanges of leaves. Planta.

[65] Abe M., Yokota K., Kurashima A., Maegawa M. (2009). High water temperature tolerance in photosynthetic activity of Zostera japonica Ascherson and Graebner seedlings from Ago Bay, Mio Prefecture, central Japan. Fish. Sci..

[66] Wang W., Vignani R., Scali M., Cresti M. (2006). A universal and rapid protocol for protein extraction from recalcitrant plant tissue for proteomic analysis. Electrophoresis.

[67] Bradford M.M. (1976). A rapid and sensitive method for the quantitation of microgram quantities of protein utilizing the principle of protein-dye binding. Anal. Biochem..

[68] George B., Kaur C., Khurdiya D.S., Kapoor H.C. (2004). Antioxidants in tomato (*Lycopersicum esculentum*) as a function of genotype. Food Chem..

[69] Vitale L., Vitale E., Guercia G., Turano M., Arena C. (2020). Effects of different light quality and biofertilizers on structural and physiological traits of spinach plants. Photosynthetica.

[70] Moulehi I., Bourgou S., Ourghemmi I., Tounsi M.S. (2017). Variety and ripening impact on phenolic composition and antioxidant activity of mandarin (*Citrus reticulate* Blanco) and bitter orange (*Citrus aurantium* L.) seeds extracts. Ind. Crop. Prod..

[71] Sun B., da Silva J.M.R., Spranger I. (1998). Factors of Vanillin Assay for Catechins and Proanthocyanidins. J. Agric. Food Chem..

